# Detection and diagnosis of rice-infecting viruses

**DOI:** 10.3389/fmicb.2013.00289

**Published:** 2013-10-09

**Authors:** Tamaki Uehara-Ichiki, Takuya Shiba, Keiichiro Matsukura, Takanori Ueno, Masahiro Hirae, Takahide Sasaya

**Affiliations:** ^1^Classification and Evaluation Research Unit, Genetic Resources Center, National Institute of Agrobiological SciencesTsukuba, Ibaraki, Japan; ^2^Plant Protection Division, Agricultural Research Center, National Agriculture and Food Research OrganizationTsukuba, Ibaraki, Japan; ^3^Kyushu Okinawa Agricultural Research Center, National Agriculture and Food Research OrganizationKoshi, Kumamoto, Japan; ^4^Food and Agricultural Materials Inspection CenterKodaira, Tokyo, Japan

**Keywords:** detection, diagnosis, rice viruses, LAR, ELISA, multiplex RT-PCR, RT-LAMP, real-time RT-PCR

## Abstract

Rice-infecting viruses have caused serious damage to rice production in Asian, American, and African countries, where about 30 rice viruses and diseases have been reported. To control these diseases, developing accurate, quick methods to detect and diagnose the viruses in the host plants and any insect vectors of the viruses is very important. Based on an antigen–antibody reaction, serological methods such as latex agglutination reaction and enzyme-linked immunosorbent assay have advanced to detect viral particles or major proteins derived from viruses. They aid in forecasting disease and surveying disease spread and are widely used for virus detection at plant protection stations and research laboratories. From the early 2000s, based on sequence information for the target virus, several other methods such as reverse transcription-polymerase chain reaction (RT-PCR) and reverse transcription-loop-mediated isothermal amplification have been developed that are sensitive, rapid, and able to differentiate closely related viruses. Recent techniques such as real-time RT-PCR can be used to quantify the pathogen in target samples and monitor population dynamics of a virus, and metagenomic analyses using next-generation sequencing and microarrays show potential for use in the diagnosis of rice diseases.

## INTRODUCTION

Rice, second only to corn in worldwide crop production, is produced in all Asian countries, in most of South and Central America, and in some of central and eastern Africa. More than 80% of the world’s rice is grown in South, East and Southeast Asian areas where the hot, humid climate favors not only rice but also the viruses and their vectors and about 30 rice viruses diseases have been reported (Abo and Sy, 1998).

In the early years of plant virus research, detection and identification of viruses were based on symptom development on infected plants or biological indexing. However, the use of symptoms for diagnosis is not reliable because symptoms vary depending on the virus strain, the presence of any mixed viral infections, the cultivar and growth stage, growing environment, and sometimes, the resemblance of viral symptoms to those induced by environmental injury. Biological assays are one of the most widely used methods among the many diagnostic techniques for plant viruses because the assays are simple and do not require special knowledge or skill (Jones, 1993; Naidu and Hughes, 2001). However, the majority of rice viruses are only transmissible by vectors, and their host range is limited to gramineous plants (Abo and Ali Fadhila, 2001). Therefore, experimental transmission of rice viruses, e.g., with mechanical or graft inoculation of indicator plants, is inconvenient and not applicable to detect or diagnosis rice viruses.

In addition, since almost all rice viruses are vector-borne, detection methods for rice viruses in their vectors, which show no noticeable symptom, have been needed to forecast and counter disease outbreaks. For these reasons, the development of serological methods based on an antigen–antibody reaction have been active areas of research, and highly sensitive and specific methods (RT-PCR, RT-LAMP, real-time RT-PCR) based on molecular or nucleic-acid techniques have recently become available. This review introduces major methods to detect target rice viruses from the 1980s and summarizes the potential of the current technologies in contributing to diagnosis of rice diseases.

## BASIC PROPERTIES OF RICE VIRUSES

The major rice viruses in Asian areas are transmitted by sucking insect vectors. Eight of these are transmitted by planthoppers or leafhoppers in a persistent manner (Hibino, 1996; Zhou et al., 2008): *Rice stripe virus* (RSV, a member of the genus *Tenuivirus*, negative sense ssRNA virus), *Rice dwarf virus* (RDV, member of *Reoviridae*, dsRNA virus), *Rice gall dwarf virus* (RGDV, a member of* Reoviridae*, dsRNA virus), *Rice ragged stunt virus* (RRSV, a member of *Reoviridae*, dsRNA virus), *Rice grassy stunt virus* (RGSV, a member of the genus *Tenuivirus*, negative sense ssRNA virus), Rice transitory yellowing virus [RTYV, same species as *Rice yellow stunt virus* (RYSV), a member of *Rhabdoviridae*, positive sense ssRNA virus; Hiraguri et al., 2010], *Rice black streaked dwarf virus* (RBSDV, a member of the* Reoviridae*, dsRNA virus) and Southern rice black streaked dwarf virus (SRBSDV, may be a member of the *Reoviridae*, dsRNA virus). Detection of these viruses in insect vectors may be easier than in rice plants, since these viruses propagate in insect bodies and the entire insect can be tested in the procedure. The tungro-disease-associated viruses, *Rice tungro bacilliform virus* (RTBV, a member of the *Caulimoviridae*, dsDNA) and *Rice tungro spherical virus* (RTSV, a member of Secoviridae, positive sense ssRNA virus) are non-propagative and transmitted in a semipersistent manner by leafhoppers, therefore, it is difficult to detect these two viruses in the insect vector using serological methods, but more sensitive methods such as real time RT-PCR and RT-LAMP can be used (Le et al., 2010; Malathi and Mangrauthia, 2013). Detection of viruses in the insect vectors is very important to forecast outbreaks and monitor disease spread because major outbreaks of insect-borne viruses are generally associated with high densities of their respective vectors. For example, we routinely detect and diagnosis RSV in its insect vector, small brown planthopper, using a commercial DAS-ELISA detection kit and a polyclonal antibody (Japan Plant Protection Association), as described later in detail.

In the Americas, *Rice hoja blanca virus* (RHBV, a member of the genus *Tenuivirus*, negative sense ssRNA virus) is the most important causal agent of viral diseases of rice and transmitted by a planthopper in a persistent manner (Hibino, 1996).

In Africa, a few viruses have been reported to infect rice plants. Rice stripe necrosis virus (RSNV, may be a member of the genus *Benyvirus*, positive sense ssRNA virus), is transmitted by the soil-inhabiting fungal pathogen *Polymyxa graminis*. The major vectors of Maize streak virus strain A (a member of genus *Mastrevirus*, ssDNA virus) are leafhoppers (Monjane et al., 2011). *Rice yellow mottle virus *(RYMV, a member of the genus *Sobemovirus*, positive sense ssRNA virus) is transmissible by mechanical inoculation and insect vectors leaf beetles (Abo and Sy, 1998; Nwilene, 1999; Banwoa et al., 2001).

After the viruses are initially transmitted to rice plants by their vectors, the rice viruses spread from the infection foci to the entire host plant through the vascular system. RSV, RDV, and RGSV propagate in the vascular tissue and mesophyll cells, but RBSDV, RGDV and RRSV are localized in the phloem and gall tissues. RTYV and RTSV propagate in the phloem tissues, and RTBV is localized in the vascular bundles (Hibino, 1996). The localization of SRBSDV, which sometimes induces confusingly similar symptoms to those caused by RBSDV infection (Zhang et al., 2008a; Zhou et al., 2008), is presumably similar to that of RBSDV in host plants.

As is the case with many plant viruses, rice viruses are known to be distributed unevenly within the host plant. In addition, as the host plants grow and tiller, some of the tillers from one individual may be infected while others may be virus-free. Therefore, samples should be taken from several parts of the entire plant body to be certain of the diagnosis.

### SEROLOGICAL DIAGNOSIS

The overview of detection methods for viruses is shown in **Table 1**. Serological methods can generally be subdivided into liquid and solid phase tests. The liquid-phase tests are the precipitin test, latex agglutination reaction (LAR), and passive hemagglutination (PHA), and solid-phase examples include the enzyme-linked immunosorbent assay (ELISA), dot-immunobinding assay (DIBA). Gel-based assay (double immunodiffusion gel assay, DIGA) have been also reported. These serological methods are used widely to detect rice viruses at pest control stations, plant protection stations, and agricultural experiment stations in Asia. Once the serological detection system is established, highly sensitive testing of a large number of samples is simpler and cheaper even though the methods are classical and several months are required to make practical antisera against rice viruses.

**Table 1:** Overview of detection methods for viruses in rice plants and in insect vectors.

Virus/family	Vectors/transmission mode	Technique
RBSDV/Reoviridae	Planthoppers/persistent	ELISA (Wang et al., 2006; Wu et al., 2013a), RT-PCR (Yang et al., 2008; Cho et al., 2013; Wu et al., 2013b), RT-LAMP (Le et al., 2010)
RDV/Reoviridae	Leafhoppers/persistent	ELISA, PHA, LAR (Omura et al., 1984), RT-PCR (Cho et al., 2013), RT-LAMP (Le et al., 2010)
RGDV/Reoviridae	Leafhoppers/persistent	DIGA (Omura et al., 1982), RT-LAMP (Le et al., 2010)
RRSV/Reoviridae	Planthoppers/persistent	ELISA (Hibino and Kimura, 1982; Luisoni et al., 1982), RT-LAMP (Le et al., 2010)
SRBSDV/Reoviridae	Planthoppers/persistent	RT-PCR (Hoang et al., 2011; Wu et al., 2013b), RT-LAMP (Zhou et al., 2012), real-time RT-PCR (Matsukura et al., 2013; Zhang et al., 2013), DIBA (Chen et al., 2012b), ELISA (Wang et al., 2012)
RGSV/*Tenuivirus*	Planthoppers/persistent	ELISA (Hibino et al., 1985; Iwasaki et al., 1985); DIBA, ELISA, LAR (Hsu et al., 1990)
RHBV/*Tenuivirus*	Planthoppers/persistent	ELISA (Marys and Carballo, 2007)
RSV/*Tenuivirus*	Planthoppers/persistent	ELISA, LAR (Omura et al., 1986; Takahashi et al., 1987), RT-PCR (Hanada et al., 1997; Cho et al., 2013; Wu et al., 2013b), RT-LAMP (Le et al., 2010), real-time RT-PCR (Zhang et al., 2008b)
RWSV/*Tenuivirus*	Planthoppers/semipersistent	ELISA (Chen and Chiu, 1989)
*RSNV/Benyvirus*	*Polymyxa graminis*	Western blot (Morales et al., 1999)
RTYV/*Rhabdoviridae*	Leafhoppers/persistent	ELISA (Takahashi et al., 1988), Western blot (Chiu et al., 1990)
RYMV*I*/*Sobemovirus*	Leaf beetles/semipersistent	ELISA (Konaté et al., 1997; Pinel et al., 2000), DIGA (Séré et al., 2005), RT-PCR (Afolabi et al., 2009)
RTSV/*Secoviridae*	Leafhoppers/semipersistent	ELISA (Bajet et al., 1985), multiplex RT-PCR (Periasamy et al., 2006), RT-LAMP (Le et al., 2010), real-time PCR (Sharma and Dasgupta, 2012)
RTBV/Caulimoviridae	Leafhoppers/semipersistent	ELISA (Bajet et al., 1985), PCR (Dasgupta et al., 1996), multiplex RT-PCR (Periasamy et al., 2006), RT-LAMP (Le et al., 2010), real-time RT-PCR (Sharma and Dasgupta, 2012)

Other important considerations for serological detection and diagnostic systems are the quality of antisera and the type of epitopes recognized (sequential or linear/conformational epitopes or continuous/discontinuous epitopes). In some cases, antisera against purified virus may contain contaminating host-plant materials, which cause non-specific reactions. In addition, the antisera may cross-react with closely related viruses. For example, the antiserum against RGSV cross-reacts with the nucleocapsid protein of RSV (Hibino et al., 1985). To reduce these undesirable non-specific or cross-reactions, antisera against a viral protein or an *Escherichina coli*-expressed viral coat protein are now being used (Chen et al., 2012b).

Antisera that recognize linear epitopes instead of conformational epitopes are chosen for immunodetection of denatured proteins. For example, target proteins that are boiled or treated with β-mercaptoethanol, can be separated by SDS-PAGE and detected in a Western blot by using antisera that recognize linear epitopes. In contrast, antisera that recognize conformational epitopes are preferred for ELISA and other methods that target proteins whose structure is preserved. In an ELISA to detect RDV in rice plants, an antiserum against intact RDV particles was more sensitive than that against RDV dissociated by SDS. But for Western blot analysis, the sensitivity of antiserum against the dissociated RDV was higher than that against intact RDV particles (Chen et al., 2012).

### LATEX AGGLUTINATION REACTION

**FIGURE 1:**
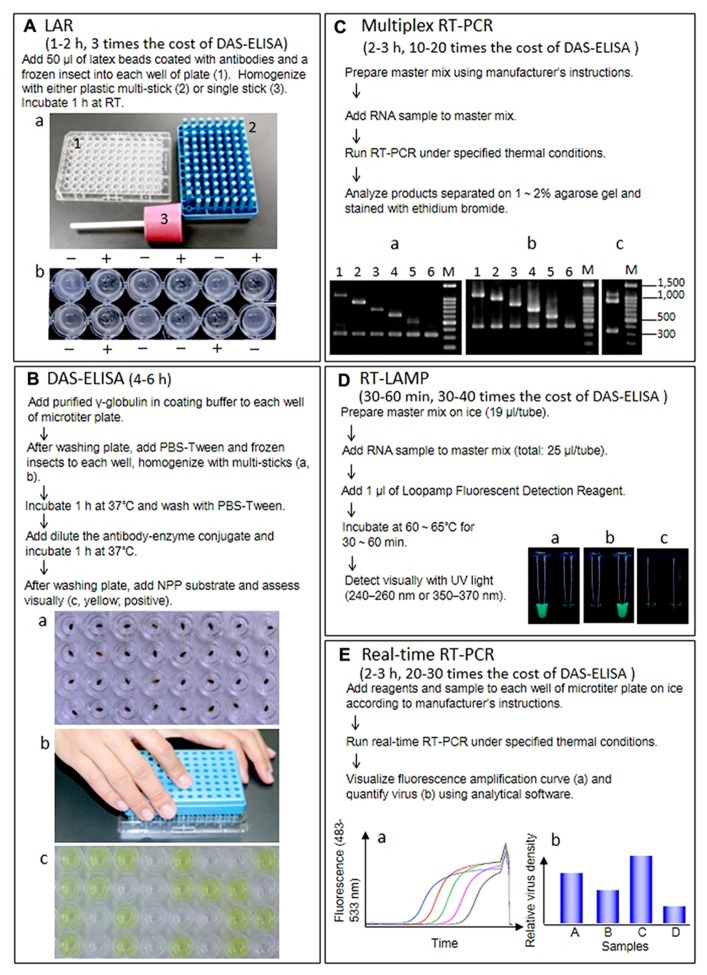
**Outline of procedure, required time, cost comparison of reagent kits per diagnosis, and results of five techniques.** **(A)** Latex agglutination reaction (LAR) to detect RSV in insect vectors. RT, room temperature. **(a)** Materials for LAR and DAS-ELISA. (1) Microtiter plate, (2) multi-sticks, (3) single stick. **(b)** Results of LAR. +, positive, -, negative. **(B)** DAS-ELISA to detect RSV in insect vectors. **(a)** The frozen insects in each well. **(b)** Homogenization of insects with multi-sticks. **(c)** Visual assessment. (Yellow; positive). **(C)** Multiplex RT-PCR to detect and distinguish 10 rice viruses. **(a)** Agarose gel electrophoresis of RT-PCR products from healthy or infected rice plants using primer set I. Lane 1, infected with RDV (1106 bp); lane 2, infected with RSV (917 bp); lane 3, infected with RBSDV(734 bp); lane 4, infected with RTYV (631 bp); lane 5, infected with RTSV (504 bp); lane 6, healthy plant (339 bp). **(b)** Agarose gel electrophoresis of RT-PCR products from healthy or infected rice plants using primer seII. Lane 1, infected with SRBSDV (1097 bp); lane 2, infected with RGDV (994 bp); lane 3, infected with RRSV (834 bp); lane 4, infected with RTBV (699 bp); lane 5, infected with RGSV (574 bp); lane 6, healthy plant (419 bp). **(c)** Agarose gel electrophoresis of RT-PCR products from rice plants infected with RDV and RSV. **(D)** RT-LAMP to detect and distinguish between SRBSDV and RBSDV. **(a)** Rice plants infected with SRBSDV. **(b)** Rice plants infected with RBSDV. **(c)** Healthy rice plants. Left, SRBSDV primer sets. Right, RBSDV primer sets. **(E)** Real-time RT-PCR to quantify SRBSDV in rice and insect vectors. **(a)** Samples with a higher density of virus yield an earlier rise in the fluorescence amplification curve, which corresponds to the density of RT-PCR products. The threshold cycle (Ct), calculated from the amplification curve, is used as an indicator of virus titer in the sample (higher virus titer results in lower Ct). **(b)** Virus titer is usually given as the relative density of virus titer to expression of a housekeeping gene (e.g., actin and ubiquitin) from the host or vector.

Latex agglutination reaction is a classical technique for immunochemical reactions in which the antigen or the antibody is attached to the surface of red blood cells or to carrier particles, e.g., latex. The virus or antibody is simply attached to the latex particles by adsorption. In PHA, virus particles or antibodies are coupled to erythrocytes by various chemical treatments. In Japan, LAR was once the most commonly used method to detect RSV in SBPHs because it is more sensitive than other precipitin assays and requires little time and minimal facilities (**Figure 1A**). But the double antibody sandwich (DAS)-ELISA in commercially available kits has started to overtake the LAR because this technique is more sensitive, yields clear results and is inexpensive (Takahashi et al., 1991)

### ELISA

Enzyme-linked immunosorbent assay is a solid-phase assay that uses antibodies labeled with enzymes that react with a substrate to yield a color change, thereby identifying the presence of a substance. We find that DAS-ELISA is easy to use for our routine diagnostic detection of RSV in insect vectors; monitoring the rate of viruliferous vector insects in early summer before rice planting season is very important to forecast rice stripe disease, an economically devastating disease, in the coming year. As shown in **Figure 1B**, up to 96 insect samples can be processed at the same time with plastic multi-sticks; for a few insect samples, we use one stick. This method is simple, and the results are robust and easy to interpret regardless of the age or sex of the insect (Uehara-Ichiki et al., 2013). Other rice viruses can also be detected with DAS-ELISA, not only in vectors but also in rice plants, and a few steps of DAS-ELISA can be skipped by grinding samples with the antibody–enzyme conjugate (Takahashi et al., 1987, 1988).

The DIBA or tissue immunoblotting assay are similar to ELISA, but in these methods, the sample extracts are spotted onto a membrane as a solid support matrix. Although false positive or false negative results are often obtained, methods to reduce the interference of chlorophyll have been reported (Srinivasan and Tolin, 1992; Chen et al., 2012a)

## NUCLEIC-ACID-BASED TECHNIQUES

Detection based on viral nucleic acids is more sensitive and specific than serological methods, and any region of a viral genome can be targeted. Rice viruses, except for RTBV, are RNA viruses, and synthesis of cDNA of the viral genome by reverse transcription (RT) is necessary before the target DNA sequence is amplified. Commercial kits to extract RNA and to synthesis cDNA from several companies such as Life Technologies, QIAGEN, Takara Biotechnologies, BIO-RAD and Promega are available. The variations of RT-PCR (e.g., multiplex RT-PCR, real-time RT-PCR) and RT-loop-mediated isothermal amplification (LAMP) have been applied to detect rice viruses from plants or insects. The commercial kits for RT-PCR are sold by the companies mentioned, and the RT-LAMP kit is sold by Eiken Chemical.

### MULTIPLEX RT-PCR

Multiplex RT-PCR uses multiple gene-specific primer sets within a single PCR mixture and can simultaneously detect two or more products in a single reaction. Therefore, the method is cost effective when two or more viruses are present in a single host plant (López et al., 2009). **Figure 1C** outlines the procedure and shows the resulting bands in the agarose gel after electrophoretic separation of the DNA fragments amplified by multiplex RT-PCR with the gene-specific primer sets (I and II), designed from coding sequences for the rice viral capsid or capsid-like proteins using the program FastPCR 6.0 (Institute of Biotechnology, University of Helsinki). Primer set I comprises two specific primers for rice actin as an internal control and 10 specific primers for RDV, RSV, RBSDV, RTYV, and RTSV, which have been reported in northeastern and eastern Asia. Primer set II comprises two specific primers for rice actin as an internal control and 10 specific primers for SRBSDV, RGDV, RRSV, RTBV, and RGSV, which have been reported in southeastern and southern Asia. Since the RT-PCR products derived from each virus differs in size, two viruses such as RDV/RSV, which are major rice viruses in Japan can be detected in a single reaction (**Figure 1C-c**). In rice fields of some countries where mixed infections with RRSV and RGSV, SRBSDV, and RSV and RBSDV, RSV and RBSDV, and RDV have been reported (Du et al., 2007; Cho et al., 2013; Wu et al., 2013b), multiplex RT-PCR is useful to rapidly and simultaneously identify the viruses.

### RT-LAMP

The LAMP technique, developed by Notomi et al. (2000), is one of the most sensitive detection methods. This method has distinctive advantages because it is highly specific for the target sequence and can be done quickly without special equipment. The specificity of LAMP is due to the recognition of six distinct sequences by four specifically designed primers, which partly alleviates the general problem of the background associated with all nucleic acid amplification methods. The LAMP reaction occurs at 60–65°C for 60 min in a water bath or heat block. We established a RT-LAMP detection system for nine rice viruses in Asia (Le et al., 2010). After SRBSDV was reported (Zhang et al., 2008a), we designed new primers derived using the S10 sequence of the SRBSDV genome for RT-LAMP using PrimerExplorerV4 () and confirmed that our RT-LAMP system could distinguish between SRBSDV and RBSDV in infected plants (**Figure 1D**). Meanwhile, Zhou et al. (2012) detected and distinguished between SRBSDV and RBSDV in host plants and insect vectors with RT-LAMP targeting S9 of the SRBSDV genome.

### REAL-TIME RT-PCR

In real-time PCR, amplification of DNA is monitored by the detection and quantitation of a fluorescent reporter signal, which increases in direct proportion to the amount of PCR product in the reaction. This technique, combined with RT, can be used to quantify target RNA. As noted already, RT-LAMP and conventional RT-PCR techniques offer rapid, sensitive, and accurate diagnosis, but they do not provide quantitative information like real-time PCR can. The starting amount of the target nucleic acid is quantitated, and the reaction can be monitored while in progress.

Real-time RT-PCR usually consists of four steps. After the first step, RT from RNA to cDNA, PCR is run using a specific primer set for the target region in the second step (usually 25–40 cycles). During this second step, fluorescence intensity, which corresponds to the amount of PCR product, is monitored for each cycle (**Figure 1E-a**). A cycle threshold (Ct), calculated from an amplification curve of fluorescence, is regarded as the virus titer in the sample (note that samples containing a higher density of virus have lower Ct values). In the third step, reaction reagents are incubated sequentially from 65 to 97°C at increments of ca. 0.1°C/s to obtain a melting curve for checking specific amplification of target region. The last step is cooling. Because virus titer is usually shown as the density of the virus relative to expression of a housekeeping gene in the same sample (**Figure 1E-b**), expression of the housekeeping gene (i.e., RNA titer transcribed from these genes in the sample) also should be determined by real-time RT-PCR. With the quantitative approach using a real-time RT-PCR for SRBSDV, the location of the virus was revealed and changes in viral density in the rice plant and subsequent effects on symptom appearance in rice can be monitored, as can virus acquisition by vector insects (Matsukura et al., 2013).

Since real-time PCR works better with small amplicons (the use of 50–200 bp is recommended), the cycling conditions for this method are shorter than for standard PCR, and the detection sensitivity is generally higher than for standard PCR assays and equivalent to that of RT-LAMP (Sharma and Dasgupta, 2012). Therefore, for the past few years, even though specific conditions and equipment are necessary for the real-time PCR system, techniques to detect and quantify rice viruses in plants and insects have been developed, revealing new insights into rice viral population dynamics (Zhang et al., 2008b, 2013; Sharma and Dasgupta, 2012; Matsukura et al., 2013).

## CONCLUSION

Advances in the technologies to detect and diagnose plant pathogens have culminated in a variety of options for researchers in laboratory and for growers. After the application of ELISA to detect plant viruses was reported (Voller et al., 1976), ELISA and its variations were utilized widely for diagnosing plant diseases. In the case of rice viruses, some reports showed that ELISA had higher sensitivity than classical methods, such as PHA and LAR, and could detect target virus in leaf extracts diluted from 10^-^^3^ to 10^-^^5^ (Hsu et al., 1990; Takahashi et al., 1991; Kawano and Takahashi, 1997).

Since PCR was devised in the late 1980s (Mullis and Faloona, 1987), the PCR and its variations have contributed to more accurate detection and identification of plant pathogens. One of the advantages of these techniques is high sensitivity compared with serological or immunological methods. For example, the sensitivity of RT-PCR was 10^2^-fold higher than dot-blot hybridization (Sharma and Dasgupta, 2012). The sensitivity of real time RT-PCR was about 10^4^-fold higher than ELISA (Zhang et al., 2008b) and 10^3^-fold higher than conventional RT-PCR (Sharma and Dasgupta, 2012). The sensitivity of RT-LAMP were about 10-fold higher than RT-PCR (Le et al., 2010; Zhou et al., 2012).

Current technologies, e.g., microarrays and next-generation sequencing also hold potential for use in diagnosing rice diseases. Microarrays are suitable techniques for high-throughput detection and identification, since an almost limitless number of pathogen probes can be placed on a single array (De Boer and López, 2012). The potential of array technology has not yet been realized because of various drawbacks that need to be resolved: sensitivity, cost, and lack of practical devices that can be used by non-technical personnel. But, it does allow us to detect and identify many pathogens including those not proven at one time (Boonham et al., 2007). Next-generation sequencing methods are being used to identify microbiomes within diseased plant tissue. This technique is largely dependent on software that can discriminate between host plant and viral sequences, but it provides new opportunities in the areas of viral candidate pathogen discovery and viral ecology (De Boer and López, 2012; Radford et al., 2012).

To select the most appropriate diagnostic methods, we need to focus on our objective. For large-scale analyses to evaluate incidence or for screening tests to monitor disease spread in fields, we should select methods using a user-friendly, evidence-based approach, an evaluation of cost per analysis and a calculation of post-test probability of disease (López et al., 2009; De Boer and López, 2012). Therefore, conventional serological methods such as LAR are still used, and ELISA remains one of the most widely applicable methods to forecast virus diseases of rice in the field.

The RT-LAMP and real-time RT-PCR techniques are too expensive for routine analysis in large surveys, but they do enable the differentiation of closely related viruses such as SRBSDV and RBSDV and very early detection of disease before symptoms are visible. Such features can help farmers and plant-health professionals to choose the best strategies to minimize potential damage. Particularly with real-time RT-PCR, applications such as screening for virus resistance and studying viral population dynamics, viral multiplication and virus–host interactions are feasible (Sharma and Dasgupta, 2012; Matsukura et al., 2013).

Conventional detection methods have only provided information on the presence of target pathogens. But progress in the development of technologies to diagnose plant diseases may not only contribute to the control of rice viral diseases, but also open opportunities to analyze potential pathogens/candidate pathogens and to develop a comprehensive understanding of the ecology of microorganisms in rice fields.

## Conflict of Interest Statement

The authors declare that the research was conducted in the absence of any commercial or financial relationships that could be construed as a potential conflict of interest.
